# Non-Invasive Prediction of Metabolic Syndrome Using Explainable Machine Learning

**DOI:** 10.3390/diagnostics16193073

**Published:** 2026-09-22

**Authors:** Islam A. Berdaweel, Sayer I. Al-Azzam, Ghaith Al-Taani, Amal Albawaana, Salam Shannag, Ameera M. Bataineh, Sireen Abdul Rahim Shilbayeh, Amjed Al Fahoum

**Affiliations:** 1 Department of Clinical Pharmacy and Pharmacy Practice, Faculty of Pharmacy, Yarmouk University, Irbid 21163, Jordan; g.altaani@yu.edu.jo (G.A.-T.); s.shannag@yu.edu.jo (S.S.); 2 Department of Clinical Pharmacy, Faculty of Pharmacy, Jordan University of Science and Technology, Irbid 22110, Jordan; 3 Faculty of Pharmacy, Department of Medicinal Chemistry and Pharmacognosy, Yarmouk University, Irbid 21163, Jordan; 4 Independent Researcher, Irbid 21110, Jordan; 5 Department of Pharmacy Practice, Pharmacy college, Princess Nourah bint Abdulrahman University, P.O. Box 84428, Riyadh 11671, Saudi Arabia; 6 Biomedical Systems and Informatics Engineering Department, Hijjawi Faculty for Engineering Technology, Yarmouk University, Irbid 21163, Jordan

**Keywords:** metabolic syndrome, machine learning, explainable artificial intelligence (XAI), non-invasive screening, Shapley Additive Explanations, predictive modeling

## Abstract

**Background**: Metabolic syndrome (MetS) is a complex health problem significantly associated with cardiovascular diseases and type 2 diabetes mellitus. Traditional diagnostic approaches rely on invasive biochemical markers, which limit their accessibility. Here, we developed an explainable machine learning (ML) framework for MetS prediction based on non-invasive demographic, anthropometric, and behavioral variables. **Method**: We conducted a retrospective observational study that included 1090 participants with MetS and 584 without MetS, using six supervised ML models, including Random Forest, AdaBoost, K-Nearest Neighbors, Bagging, Logistic Regression, and Ensemble learning. We trained and validated these models using stratified 10-fold cross-validation. Predictive performance was evaluated using accuracy, precision, recall, specificity, negative predictive value, area under the receiver operating characteristic curve (AUC) and F1-score. Shapley Additive Explanations (SHAP) applied to the Random Forest classifier evaluated interpretability. A 1:1 balanced sensitivity analysis was subsequently performed using the same leakage-safe predictor set and validation framework to assess whether model performance was materially influenced by outcome prevalence. **Results**: In the natural-prevalence primary cohort (*n* = 1674; 65.1% with MetS), leakage-safe models showed moderate discrimination. Ensemble achieved the highest mean AUC (0.751, 95% CI 0.731–0.771), followed by Random Forest (0.745, 95% CI 0.723–0.767) and Bagging (0.742, 95% CI 0.725–0.760). SHAP analysis identified (body mass index) BMI, age, and body weight as the dominant contributors, with additional contributions from physical activity, and dietary and sociodemographic variables. In the 1:1 balanced sensitivity cohort (*n* = 1000), discrimination remained similar, with Ensemble achieving the highest mean AUC (0.742, 95% CI 0.704–0.780). **Conclusion**: Explainable ML models based on accessible non-laboratory variables provided moderate discrimination of prevalent MetS and may serve as candidate pre-laboratory risk-stratification tools. They should not replace established diagnostic criteria, and external validation, recalibration, and prospective evaluation of clinically relevant thresholds are required before implementation.

## 1. Introduction

According to the National Cholesterol Education Program Adult Treatment Panel III (NCEP ATP III) criteria, metabolic syndrome (MetS) is defined as the presence of at least three of the following five components: abdominal obesity, elevated triglycerides, reduced high-density lipoprotein (HDL) cholesterolcholesterol, elevated blood pressure, and elevated fasting glucose [1]. MetS reflects a systemic metabolic imbalance driven by interactions between genetic predisposition, behavioral risk factors, chronic inflammation, sedentary lifestyle, and environmental influences [2,3]. Recently, the global burden of MetS has risen considerably, with estimates indicating that approximately one-quarter to one-third of adults suffer from MetS depending on the diagnostic criteria used, including NCEP-ATP III, International Diabetes Federation (IDF), and harmonized criteria. However, the MetS prevalence varies substantially with ethnicity, age, sex, socioeconomic status, and diagnostic definition [4,5]. Notably, the Middle East and North Africa (MENA) region has one of the highest reported prevalences of MetS worldwide, with estimates reaching up to 63% among adults [2,6,7,8]. This high burden highlights the urgent need for accessible screening strategies in this region. Importantly, MetS is not only a collection of isolated risk factors but rather is a synergistic metabolic state that is associated with increased risk of several cardiovascular diseases (CVD) and cardiovascular (CV)-related mortality. Also, the co-occurrence of obesity, hypertension, and insulin resistance in one individual increases the risk for progression to type 2 diabetes mellitus (T2DM)and chronic vascular dysfunction [9,10]. Consequently, early identification of individuals at increased metabolic risk has become an important clinical priority.

The NCEP ATP III proposed standardized clinical criteria for metabolic syndrome [11]. Although these criteria provide clinical standardization, their application depends partly on invasive laboratory tests, including fasting plasma glucose, triglyceride, and high-density lipoprotein cholesterol levels. These diagnostic requirements often involve blood sampling with laboratory processing, and clinical attendance, in addition to considerable healthcare costs. These requirements limit patients’ accessibility and delay early risk detection. Such barriers are particularly relevant in settings with limited medical infrastructure, and for individuals with limited access to healthcare [11,12]. Additionally, socioeconomic status, and reluctance to undergo invasive procedures may further delay diagnosis, resulting in under-screening of metabolically susceptible individuals. MetS often progresses as a silent medical condition in its early stages until clinically significant complications develop, resulting in missed opportunities for early interventions [13,14,15,16].

Currently, there is a growing scientific interest in developing predictive strategies for early MetS identification using easily available, non-invasive measures, such as anthropometric indices, behavioral factors, medical history, lifestyle and dietary behaviors, which are increasingly being explored as potential predictors for computational risk modeling [17,18]. Advances in artificial intelligence (AI) and machine learning (ML) have supported the development of predictive frameworks for metabolic risk estimation. ML algorithms are able to identify multidimensional dependencies, nonlinear and complex interactions among clinical and behavioral variables, compared to traditional statistical approaches that often rely on linear assumptions among predictors [17,19,20,21].

Although several studies have reported promising models with predictive performance for MetS classification using ML approaches, they suffer from methodological limitations that may hinder their generalizability and clinical applicability, highlighting the need for more robust and reliable predictive models. A key limitation of multiple published studies is their reliance on relatively small sample sizes, self-reported information or highly selected populations, which increases the risk of selection, recall, and reporting biases, limits the representativeness of their findings, and may result in residual confounding due to inadequate adjustment for important demographic, clinical, lifestyle, and socioeconomic factors [19,20,22,23,24]. Furthermore, some of these studies have utilized imbalanced datasets, which can bias model learning toward the majority subgroup and reduce sensitivity for detecting true MetS cases [25,26]. In some cases, predictive performance was dependent on invasive biomarkers such as fasting glucose, triglycerides, and HDL cholesterol levels, which limits the applicability of the resulting model in non-invasive screening settings [18,20,26]. Additionally, heterogeneity in validation procedures and performance reporting among different studies prevents direct comparisons and raises concerns regarding the reproducibility and robustness of these models [26,27]. Model complexity and limited interpretability are additional important limitations. Clinicians are more likely to adopt decision-support systems when the prediction rationale can be explained and aligned with established medical understanding. Several ML systems studied for MetS function as complex “black-box” models in which the internal reasoning behind predictions remains insufficiently transparent for clinicians and healthcare providers. These studies have utilized complex models that need a relatively large number of features, making them difficult to apply and to use to interpret the predicted results despite high performance and predictive accuracy [22,28,29].

Explainable artificial intelligence (XAI) techniques have recently emerged as important tools for improving transparency in healthcare ML systems. Among available explainability methods, Shapley Additive Explanations (SHAP) have gained increasing attention due to their ability to quantify the contribution of individual predictors toward model outputs while supporting both global and patient-specific interpretation [30,31]. SHAP enables identification of influential risk factors, visualization of nonlinear predictor effects, and individualized explanation of clinical predictions, thereby improving interpretability, model transparency and supporting clinical interpretability. Such approaches may be particularly valuable in MetS prediction because risk emerges from interactions among multiple demographic, behavioral, and physiological characteristics rather than isolated clinical variables [31,32]. Several studies have applied explainable ML with SHAP for non-invasive MetS prediction but have not consistently tested whether the reported performance metrics remain valid when predictor variables overlap with MetS diagnostic criteria such as hypertension, dyslipidemia and diabetes.

To address these limitations, the present study predefined a leakage-safe predictor set by excluding variables that directly represented or closely overlapped with the NCEP ATP III diagnostic criteria. We hypothesized that ML models trained exclusively on readily available, non-invasive demographic, anthropometric, and behavioral variables could identify individuals with MetS with clinically useful predictive performance. We further hypothesized that removing predictors overlapping with the NCEP ATP III diagnostic criteria would provide a more robust and clinically applicable estimate of model performance while retaining meaningful predictive information.

## 2. Materials and Methods

### 2.1. Data Source and Study Population

#### 2.1.1. Setting

This study was conducted between September 2012 and December 2013, in the outpatient clinics of four major public hospitals representing diverse geographic regions of Jordan, including King Abdullah University Hospital (KAUH) Institutional Review Board (IRB) committee, IRB reference no 5/2012, 22 May 2012), Princess Basma Hospital in northern Jordan, Al-Basheer Hospital in central Jordan, and Al-Karak Hospital in southern Jordan with ethical approval obtained from the Ministry of Health of Jordan IRB committee for these three hospitals (IRB reference no: 5842, 2 July 2012). All patient data were anonymized, and the study adhered to the Declaration of Helsinki guidelines for ethical research involving human participants.

#### 2.1.2. Data Collection

All adults who were attending outpatient clinics within the participating hospitals during the study duration were eligible to participate. Participants were excluded if they had Alzheimer’s disease, were pregnant, had secondary causes of metabolic abnormalities (e.g., Cushing’s syndrome, hypothyroidism), were unable to communicate in Arabic, or were unable to provide informed consent. A trained research pharmacist (PharmD) conducted structured interviews with eligible participants after explaining the study objectives and obtaining written informed consent from each participant. A structured data collection instrument was utilized to collect participants’ demographics (age, gender, educational level, occupation, marital status, and health-insurance status), smoking, physical activity, body weight, body mass index, waist circumference, comorbidities and medical history (e.g., hypertension, diabetes, dyslipidemia, other chronic conditions) and current medication use. Dietary habits were recorded according to the reported number of days per week characterized by healthy and high-fat dietary patterns (e.g., “Healthy diet days/week” and “High-fat diet days/week”), with responses ranging from 0 to 7 days per week. A total of 1676 participants met the eligibility criteria and consented to participate.

#### 2.1.3. Metabolic Syndrome Classification

Participants were assessed for MetS using the collected clinical and laboratory data, consistent with the NCEP ATP III diagnostic framework. MetS was defined as having at least three of the following diagnostic criteria: abdominal obesity (waist circumference > 102 cm in men; >88 cm in women), elevated triglycerides (TG ≥ 150 mg/dL (1.7 mmol/L), or on triglyceride-lowering therapy), reduced high-density lipoprotein (HDL < 40 mg/dL in men; <50 mg/dL in women; or on HDL-raising therapy), elevated blood pressure (≥130/85 mmHg, or on antihypertensive therapy), and elevated fasting glucose (≥100 mg/dL (5.6 mmol/L), or on antidiabetic therapy). Although MetS classification relied on laboratory and clinical measurements consistent with NCEP ATP III criteria, these values were used solely to establish the outcome variable and were excluded from the machine learning predictor set.

### 2.2. Study Design

This retrospective observational study investigated the feasibility of predicting MetS using exclusively non-invasive demographic, anthropometric, and behavioral variables. A structured ML framework was designed to determine whether easily obtainable patient characteristics could reliably discriminate between individuals with and without MetS without dependence on laboratory biomarkers or biochemical testing. To ensure a transparent and reproducible workflow, we followed a structured analytical pipeline that included data acquisition, eligibility screening, data cleaning, missing data handling, feature preparation, ML model development, stratified cross-validation, comparative performance evaluation, and SHAP-based interpretability analysis.

### 2.3. Data Preprocessing and Cohort Construction

The source dataset contained 1676 participants. Following verification of the metabolic syndrome outcome, 1674 participants had an evaluable MetS criterion-count outcome and were included in the primary analysis. Two participants were excluded because the MetS outcome was missing or unusable. The final primary cohort comprised 1090 participants with MetS and 584 without MetS, corresponding to an observed MetS prevalence of 65.1%. No participants with an evaluable outcome were removed for the purpose of class balancing in the primary analysis.

Missing predictor values were handled within the model-development framework rather than by complete-case deletion. For each outer cross-validation iteration, numeric missing values were imputed using medians estimated exclusively from the corresponding outer training partition. Categorical variables were encoded using category levels derived from the same training data. Numeric predictors were then standardized using z-score normalization, with the mean and standard deviation estimated from the outer training partition and applied unchanged to the corresponding held-out test fold.

Continuous predictors were retained as numeric variables, whereas categorical predictors were transformed into machine-readable encoded variables. All preprocessing parameters were therefore estimated only from training data within each outer cross-validation iteration, preventing information from the held-out test fold from influencing preprocessing or model development. The outcome was binary, with MetS coded as 1 and absence of MetS as 0.

### 2.4. Predictor Variables

To minimize outcome–predictor circularity, the primary predictive models used a predefined set of 12 accessible, non-laboratory predictors that did not directly reproduce the NCEP ATP III diagnostic criteria. These included age, sex, body mass index (BMI), body weight, marital status, education level, occupation, health-insurance status, smoking status, physical-exercise frequency, healthy-diet frequency, and high-fat-diet frequency.

Variables directly representing or closely encoding components of the MetS diagnostic framework were excluded from the primary predictor matrix. These included laboratory measurements and physician-assigned cardiometabolic diagnoses or related treatment variables, such as hypertension, diabetes mellitus, dyslipidemia, cardiovascular disease, and medication use.

Waist circumference was also excluded because abdominal adiposity is directly incorporated into the NCEP ATP III definition. The resulting predictor set was therefore designed to evaluate the feasibility of pre-laboratory risk stratification rather than prediction based on variables that could reproduce the diagnostic outcome.

### 2.5. ML Models

For a complete evaluation of the prediction performance, several supervised ML algorithms with different computational assumptions were implemented, such as Random Forest (RF), Adaptive Boosting (AdaBoost), K-Nearest Neighbors (KNN), Bootstrap Aggregating with Out-of-Bag estimation (Bagging-OOB), Logistic Regression (LR), and an Ensemble learning classifier that combines complementary models.

### 2.6. Model Training and Validation

Model development and performance estimation were conducted using a nested stratified cross-validation framework to minimize optimistic bias and ensure that hyperparameter tuning remained independent of final performance assessment. The complete outcome-evaluable cohort was entered into an outer stratified 10-fold cross-validation procedure while preserving the observed MetS class distribution within each fold.

At each outer iteration, one fold was held out exclusively as the test fold for performance estimation, whereas the remaining nine folds constituted the outer training set. No information from the outer test fold was used during preprocessing, feature transformation, model fitting, or hyperparameter selection. All preprocessing parameters, including numeric imputation, categorical encoding, and z-score standardization, were estimated using the outer training data and then applied unchanged to the corresponding held-out test fold.

Hyperparameter optimization was performed within each outer training set using an inner stratified 5-fold cross-validation procedure. Model-specific candidate configurations were evaluated only within these inner folds, and the hyperparameter combination yielding the best prespecified optimization performance was selected. The selected configuration was then refitted using the complete outer training set and applied once to the held-out outer test fold.

This procedure was repeated across all 10 outer folds so that every participant contributed once to out-of-sample test-set prediction. The resulting predictions were stored as common out-of-fold records and were used to estimate the final classification and discrimination performance of each model. Accuracy, sensitivity, specificity, positive predictive value, negative predictive value, F1-score, area under the receiver operating characteristic curve, and related performance measures were calculated from these outer-fold test results. Fold-specific estimates were retained to summarize mean performance, variability, and 95% confidence intervals.

The same nested validation structure was applied consistently to Random Forest, AdaBoost, K-Nearest Neighbors, Bagging, Ensemble learning, and Logistic Regression. A classification probability threshold of 0.50 was used for the primary threshold-dependent comparisons. This nested design ensured that the outer held-out fold functioned solely as an independent test fold for performance estimation, while model selection and tuning were confined to the corresponding outer training data.

### 2.7. Statistical Analysis and Performance Evaluation

Mean ± standard deviation (SD) or median were used to describe the continuous variables, and percentage for categorical variables. Independent sample *t*-tests were applied for between-group comparisons for normally distributed continuous variables, Mann–Whitney U tests for non-normally distributed continuous variables and chi-square tests for categorical variables. A two-tailed *p*-value of less than 0.05 was considered statistically significant.

Complementary classification metrics were applied for ML performance evaluation, including accuracy, recall (sensitivity), specificity, precision (positive predictive value), negative predictive value (NPV), F1-score, and AUC. Since reliance on a single performance metric may be misleading, discrimination performance was evaluated using receiver operating characteristic (ROC) curves, precision–recall curves, calibration plots, confusion matrices, and decision-curve analysis (DCA) to assess clinical usefulness.

The ML analyses, performance evaluation, calibration analyses, and computational runtime measurements were conducted in MATLAB R2024b and model explainability analyses were performed using the SHAP package (version 0.46). Statistical analyses of baseline characteristics were conducted in IBM SPSS Statistics version 29.0 (IBM Corp., Armonk, NY, USA).

### 2.8. Explainability and SHAP-Based Interpretability

Shapley Additive Explanations (SHAP) were used to interpret the Random Forest model at both global and individual prediction levels. SHAP analysis was performed using the final leakage-safe predictor matrix and the corresponding fitted model predictions. Global feature importance was summarized using mean absolute SHAP values, while local SHAPs were used to illustrate how individual predictors contributed to representative predictions. SHAP values were interpreted as model-attribution measures and not as causal effects.

### 2.9. Sensitivity Analysis

To evaluate whether model performance was substantially influenced by the observed outcome prevalence, a separate 1:1 balanced sensitivity analysis was conducted. The balanced cohort included 1000 participants, comprising 500 participants with MetS and 500 without MetS, selected through reproducible undersampling of the larger outcome class.

The same 12 leakage-safe predictors, six classification algorithms, preprocessing procedures, hyperparameter optimization, and stratified 10-fold outer cross-validation framework were applied. Performance estimates from the balanced cohort were compared with those obtained from the natural-prevalence primary analysis. The balanced analysis was considered a sensitivity analysis and did not replace the primary natural-prevalence estimates.

## 3. Results

### 3.1. Study Population and Baseline Characteristics

Of the 1676 source records, 1674 participants had an evaluable metabolic syndrome (MetS) outcome and entered the primary analysis (Figure 1). The cohort included 1090 participants with MetS and 584 without MetS, corresponding to an observed MetS prevalence of 65.1%. The two remaining source records lacked a usable MetS outcome. No outcome-evaluable participant was removed to impose a 1:1 class distribution in the primary analysis. Baseline characteristics are summarized in Table 1.

The primary model was restricted to 12 prespecified non-laboratory predictors: age, sex, BMI, body weight, marital status, education, occupation, health-insurance status, smoking status, physical-exercise frequency, healthy-diet frequency, and high-fat-diet frequency. Laboratory measurements, medication variables, physician-assigned cardiometabolic diagnoses, and variables directly representing MetS diagnostic components were excluded before model fitting.

### 3.2. Primary Machine-Learning Performance in the Naturally Distributed Cohort

Table 2 and Figure 2 summarize fold-averaged performance from the outer stratified 10-fold cross-validation. Discrimination was moderate across the evaluated classifiers, while operating characteristics at the prespecified 0.50 threshold differed substantially.

The Ensemble classifier had the highest mean AUC, 0.751 (95% CI 0.731–0.771), followed by Random Forest at 0.745 (0.723–0.767) and Bagging at 0.742 (0.725–0.760). Logistic Regression achieved an AUC of 0.728 (0.705–0.751), while KNN and AdaBoost achieved mean AUCs of 0.709 and 0.704, respectively.

Random Forest provided one of the more balanced threshold-dependent profiles: accuracy of 72.22%, sensitivity of 77.25%, specificity of 62.86%, PPV of 79.63%, NPV of 59.74%, and F1-score of 78.35%. Bagging was similar, with accuracy of 71.87%, sensitivity of 76.97%, specificity of 62.35%, and F1-score of 78.04%. The Ensemble model traded sensitivity for specificity at the 0.50 threshold, with sensitivity of 63.39% and specificity of 72.09%. Logistic Regression was comparatively balanced, with sensitivity of 68.44% and specificity of 65.25%.

KNN produced the highest sensitivity, 94.50%, but specificity was only 19.83%, creating a large false-positive burden. AdaBoost showed the opposite threshold behavior. Its mean AUC remained approximately 0.70, yet mean sensitivity at 0.50 was only 5.05% while specificity was 98.63%. The out-of-fold probabilities for AdaBoost were generally below 0.50 even though they ranked MetS-positive participants better than chance, explaining the divergence between threshold-independent AUC and classification at a fixed threshold.

### 3.3. Calibration and Decision-Curve Analysis

Calibration differed materially between algorithms (Table 3 and Figure 3). Random Forest had a calibration intercept of 0.532, slope of 0.915, and the lowest primary-analysis Brier score of 0.195. Bagging showed a similar slope of 0.917 and Brier score of 0.198. Logistic Regression had a slope close to unity (1.032), although its intercept was 0.612. The Ensemble model had an intercept of 0.950, slope of 1.402, and Brier score of 0.221.

KNN and AdaBoost showed the greatest calibration concerns. KNN had a calibration slope of 0.074 despite a Brier score of 0.204. AdaBoost had an intercept of 1.313, slope of 0.047, and Brier score of 0.504. These raw probability estimates should therefore not be interpreted as calibrated absolute MetS probabilities without further recalibration and external validation.

Decision-curve analysis demonstrated that net clinical benefit varied across threshold probabilities and differed among classifiers. The relative clinical utility of the models therefore depended on the selected decision threshold rather than following the AUC ranking alone. The treat-all strategy declined as the threshold probability increased, whereas the model-based strategies showed threshold-dependent net benefit (Figure 3). These findings support evaluation of threshold-specific clinical utility rather than selection of a model based on discrimination alone.

### 3.4. Confusion-Matrix Analysis and Numerical Consistency

Pooled out-of-fold confusion matrices reinforce the model-specific operating differences (Figure 4). Random Forest classified 842 MetS cases correctly and missed 248, with 367 true-negative and 217 false-positive classifications. Bagging produced 839 true positives, 251 false negatives, 364 true negatives, and 220 false positives. The Ensemble model reduced false positives to 163 but increased false negatives to 399.

KNN identified 1030 of 1090 MetS-positive participants but generated 468 false positives among 584 non-MetS participants. AdaBoost generated only 55 true positives and 1035 false negatives, while correctly classifying 576 of 584 non-MetS participants. These pooled counts are consistent with the threshold-dependent patterns reported above.

### 3.5. Predictor Redundancy and Explainability

Formal redundancy assessment did not identify severe multicollinearity. BMI had the highest variance inflation factor (VIF = 4.046), followed by body weight (VIF = 3.983). Every other encoded predictor had VIF below 2; therefore, no predictor exceeded the prespecified VIF threshold of 5.

Random Forest SHAP analysis, performed only on the final leakage-safe predictor matrix, identified BMI as the dominant global contributor, followed by age and body weight. Physical exercise and dietary-frequency variables also contributed to predictions, together with sex, education, occupation, marital status, insurance status, and smoking. No laboratory measurement, physician-assigned hypertension, diabetes, dyslipidemia, cardiovascular diagnosis, or medication variable appears in the explanation analysis (Figure 5).

SHAP values explain how the fitted model allocated predictive contribution among features. They should not be interpreted as causal effects or as evidence that modifying a given predictor would produce a corresponding change in MetS status.

### 3.6. Subgroup Performance

Discrimination varied across age and BMI strata (Figure 6). For Random Forest, AUC was 0.677 (95% CI 0.605–0.747) among participants aged <45 years, 0.693 (0.653–0.731) among those aged 45–59 years, and 0.738 (0.689–0.785) among those aged ≥60 years. The Ensemble classifier reached the highest observed age-stratum AUC, 0.763 (0.717–0.807), among participants aged ≥60 years.

Random Forest AUC also varied by BMI: 0.694 (0.629–0.760) for BMI < 25 kg/m^2^, 0.747 (0.705–0.785) for BMI 25.0–29.9 kg/m^2^, and 0.592 (0.543–0.639) for BMI ≥ 30 kg/m^2^. Similar heterogeneity was present for other classifiers. These exploratory results argue against assuming that aggregate discrimination applies uniformly across demographic or anthropometric strata.

### 3.7. Balanced-Cohort Sensitivity Analysis

A separate sensitivity analysis evaluated whether the primary findings were driven by the observed 65.1% MetS prevalence. Reproducible 1:1 undersampling produced a cohort of 1000 participants, with 500 MetS-positive and 500 MetS-negative participants. The same 12 leakage-safe predictors and validation framework were used.

Discrimination remained in the same general range as in the natural-prevalence analysis (Table 4). Mean AUC was 0.742 for Ensemble, 0.732 for Bagging, 0.728 for Random Forest, and 0.711 for Logistic Regression. KNN and AdaBoost achieved mean AUCs of 0.700 and 0.698, respectively. Equalizing class frequencies therefore did not materially improve discrimination, indicating that class prevalence alone was not responsible for the performance observed in the leakage-safe models.

Threshold-dependent patterns also persisted. Bagging provided sensitivity of 75.8% and specificity of 64.8%; Random Forest achieved 72.6% and 62.8%, respectively. Ensemble specificity increased to 79.6% while sensitivity fell to 49.0%. AdaBoost again showed 5.0% sensitivity and 98.6% specificity at the 0.50 threshold. Calibration was more favorable for Random Forest, Bagging, KNN, and Logistic Regression in the balanced cohort, whereas AdaBoost remained poorly calibrated.

### 3.8. Computational Performance

Training time varied more than prediction time (Table 5). In the primary analysis, mean training time per outer fold ranged from 1.95 s for Logistic Regression to 82.02 s for the Ensemble classifier. Mean prediction time ranged from 0.00083 s to 0.150 s per held-out fold. In the balanced sensitivity analysis, mean training time ranged from 1.65 s for Logistic Regression to 75.70 s for Ensemble, while prediction time ranged from 0.00068 s to 0.161 s.

## 4. Discussion

### 4.1. Principal Findings

The present study evaluated whether metabolic syndrome (MetS) could be discriminated against using readily accessible demographic, anthropometric, behavioral, and sociodemographic variables while avoiding predictors that directly reproduce or closely encode the diagnostic definition of MetS. In the natural-prevalence cohort, the highest mean AUC was 0.751 for Ensemble, followed by Random Forest and Bagging. Balancing the classes did not materially improve discrimination. These findings support the use of accessible non-laboratory variables for candidate pre-laboratory risk stratification, while indicating that such models should not be considered substitutes for established diagnostic assessment.

No classifier was uniformly superior across performance measures. Ensemble achieved the highest mean AUC but lower sensitivity at the prespecified threshold, whereas Random Forest and Bagging provided more balanced sensitivity and specificity. KNN achieved high sensitivity at the cost of low specificity, while AdaBoost demonstrated moderate threshold-independent discrimination but very low sensitivity at the 0.50 threshold. These findings illustrate the importance of evaluating discrimination together with threshold-dependent performance when considering models for potential screening applications.

This is supported by previous work showing that non-invasive ML models can identify MetS, although performance varies substantially according to the predictor set and validation strategy. For example, a large study using non-invasive characteristics reported useful discrimination, while studies incorporating biochemical and cardiometabolic variables have generally reported higher AUCs [33,34].

No single classifier was superior across all performance dimensions. Although Ensemble produced the highest threshold-independent discrimination, Random Forest and Bagging demonstrated more favorable sensitivity–specificity trade-offs at the prespecified 0.50 threshold. K-Nearest Neighbors (KNN) achieved the highest sensitivity (94.50%) but with very low specificity (19.83%), whereas AdaBoost showed the opposite pattern, with very high specificity (98.63%) but sensitivity of only 5.05%. These findings emphasize that model selection cannot reasonably be based on AUC alone. Contemporary guidance for clinical prediction models recommends considering discrimination together with calibration and clinical utility, because a model that ranks individuals reasonably well may still perform poorly when used at a particular decision threshold [35].

### 4.2. Predictor Selection and Outcome–Predictor Circularity

A central methodological consideration in MetS prediction is the relationship between the predictors and the outcome definition itself. Under the NCEP ATP III framework, MetS is defined by the presence of at least three of five components: abdominal obesity, elevated triglycerides, reduced HDL cholesterol, elevated blood pressure, and elevated fasting glucose. Consequently, laboratory measurements of glucose and lipids, as well as physician-assigned diagnoses such as hypertension, diabetes, and dyslipidemia and their related treatments, may contain information that is already embedded in or closely linked to the outcome. Models incorporating such variables may therefore achieve high discrimination while addressing a clinically different question from pre-laboratory risk stratification.

The present predictor framework was therefore intentionally restricted to 12 accessible variables that did not directly reproduce the ATP III diagnostic components. This restriction resulted in a lower level of discrimination than that observed in models incorporating clinically derived or biochemical variables, but this reduction should be interpreted in the context of the prediction task rather than as evidence of inadequate algorithmic performance. Reported performance of MetS prediction models varies considerably across studies, reflecting differences in predictor selection and methodological approaches. A systematic review of 11 MetS prediction-model studies identified 48 candidate predictors and found substantial heterogeneity in model development and validation methods, with reported c-statistics ranging from 0.67 to 0.95. Importantly, the review noted that several predictors were themselves components of the MetS outcome definition, highlighting the potential for optimistic performance estimates when predictors overlap with the outcome [16].

Importantly, the balanced sensitivity analysis did not materially improve discrimination, with the Ensemble model achieving an AUC of 0.742 compared with 0.751 in the natural-prevalence cohort. This finding suggests that the moderate discrimination observed in the primary analysis was not simply attributable to the unequal outcome distribution.

### 4.3. Discrimination, Thresholds, and False-Positive Burden

The threshold-dependent results further demonstrate why discrimination, and classification should be interpreted separately. At the prespecified probability threshold of 0.50, Random Forest achieved sensitivity of 77.25% and specificity of 62.86%, while Ensemble achieved higher specificity (72.09%) at the expense of lower sensitivity (63.39%). KNN provided substantially greater case detection, with sensitivity of 94.50%, but its specificity of 19.83% indicates that this operating point would generate a considerable number of false-positive classifications. In contrast, the threshold-dependent performance of AdaBoost illustrates that a model can have moderate ranking ability, reflected by an AUC of approximately 0.70, while performing poorly at a particular probability threshold.

The apparent discrepancy between AdaBoost’s AUC and its very low sensitivity at a threshold of 0.50 is therefore expected rather than contradictory. AUC summarizes discrimination across the full range of possible thresholds, whereas sensitivity and specificity describe performance at a single operating point. Accordingly, the 0.50 threshold should be regarded as a standardized comparison threshold rather than a clinically optimized decision threshold. In a future clinical application, the threshold should be selected prospectively according to the intended use of the model, the relative consequences of missed MetS cases and unnecessary referrals, and the capacity available for subsequent confirmatory assessment. Decision-curve analysis provides a framework for incorporating such clinical consequences by evaluating net benefit across clinically relevant threshold probabilities.

### 4.4. Calibration and Clinical Utility

Calibration provided information that was not captured by discrimination alone. Random Forest and Bagging demonstrated calibration slopes close to 1 and the lowest Brier scores in the primary analysis, although their positive calibration intercepts indicate residual systematic miscalibration. Logistic Regression also had a slope close to 1, whereas Ensemble showed a steeper slope and KNN and AdaBoost demonstrated substantial distortion of the predicted probability scale. These findings reinforce the importance of evaluating both discrimination and calibration when prediction models are intended to generate individual risk estimates. Calibration should not be inferred from the calibration slope alone, and assessment of both calibration-in-the-large and calibration slope, preferably together with graphical assessment, provides a more complete evaluation of predictive probabilities [36].

Accordingly, the predicted probabilities generated by the current models should not yet be interpreted as calibrated individual probabilities of MetS. External validation in an independent population is required to determine whether systematic miscalibration persists and whether population-specific recalibration is necessary. This issue is particularly relevant because outcome prevalence and case mix can differ between development and implementation populations, potentially affecting both calibration and clinical utility. Current prediction-model guidance emphasizes external validation and assessment of discrimination, calibration, and clinical usefulness before a model is considered for clinical implementation [37].

The decision-curve analysis similarly indicated that the relative net benefit of the models varied across threshold probabilities rather than following the AUC ranking consistently. Thus, the clinical usefulness of a particular classifier cannot be established from discrimination alone and will depend on the threshold at which the model is intended to trigger confirmatory assessment. Because the present study does not include prospective evaluation of a predefined clinical pathway, the DCA findings should be regarded as exploratory rather than as evidence for implementation at a specific threshold [38].

### 4.5. Explainability and Predictor Redundancy

The SHAP analysis provided an interpretable description of how the Random Forest model used the available predictors. BMI, age, and body weight were the dominant contributors, followed by physical activity, dietary variables, smoking, sex, and sociodemographic characteristics. The prominence of anthropometric variables is consistent with the established relationship between excess adiposity and the metabolic abnormalities that constitute MetS and is also consistent with previous non-invasive ML studies in which age and anthropometric characteristics have contributed substantially to model predictions [34]. However, SHAP values represent feature contributions within the fitted predictive model and should not be interpreted as causal effects. In particular, a large SHAP contribution does not demonstrate that modifying that predictor would produce a corresponding reduction in MetS risk. SHAP is therefore useful for describing model behavior and improving transparency, but causal interpretation requires a different analytical framework. The use of SHAP for global and local model interpretation is consistent with its original formulation as an additive feature-attribution framework.

BMI and body weight also showed the highest VIF values in the predictor matrix (4.046 and 3.983, respectively), reflecting their expected anthropometric overlap. Nevertheless, both remained below the prespecified VIF threshold of 5, and no predictor demonstrated severe multicollinearity according to the applied criterion. Their retention therefore appears reasonable for the present model, although a more parsimonious anthropometric representation may be preferable in future work if external validation demonstrates that comparable discrimination can be maintained with fewer correlated variables.

### 4.6. Subgroup Heterogeneity

Model discrimination was not uniform across age and BMI strata. For Random Forest, AUC increased across the prespecified age groups, from 0.677 among participants aged <45 years to 0.738 among those aged ≥60 years, while performance was lower among participants with BMI ≥ 30 kg/m^2^ (AUC 0.592) than among those with BMI 25.0–29.9 kg/m^2^ (AUC 0.747). One possible explanation is that anthropometric characteristics may provide less discriminatory information among individuals with established obesity because elevated adiposity is more common within this subgroup; however, the present data cannot establish the mechanism underlying these differences.

These subgroup findings should therefore be interpreted cautiously because the study was not specifically powered to detect differences in predictive performance between strata, and confidence intervals were wider within some subgroups. Nevertheless, the observed heterogeneity highlights the importance of evaluating model performance across clinically relevant populations rather than assuming that aggregate discrimination applies uniformly to all individuals. External validation should consequently examine discrimination, calibration, and clinical utility across age, sex, BMI, and other relevant population strata. Such subgroup evaluation is consistent with current recommendations for transparent reporting and evaluation of clinical prediction models [35].

### 4.7. Sensitivity Analysis and Robustness

The 1:1 balanced sensitivity analysis produced broadly similar overall discrimination to the natural-prevalence primary analysis. Ensemble remained the highest-performing model by AUC (0.742, 95% CI 0.704–0.780), followed by Bagging (0.732, 95% CI 0.697–0.768) and Random Forest (0.728, 95% CI 0.694–0.762). Although the exact model ranking was not identical to that of the primary analysis, the overall level of discrimination remained comparable. These findings support the use of the natural-prevalence cohort as the principal analysis because it retains all outcome-evaluable participants and reflects the observed prevalence of MetS.

The balanced analysis nevertheless provides a useful robustness assessment because class distribution can influence threshold-dependent metrics and model training. However, undersampling discards observations and creates an artificial outcome prevalence; consequently, estimates of predictive values and probability calibration from the balanced cohort should not be interpreted as estimates of performance in the underlying study population. Previous work has demonstrated that data-balancing strategies can materially influence MetS prediction performance, supporting the value of explicitly examining the effect of class distribution rather than relying on a single sampling strategy.

### 4.8. Potential Application and Computational Feasibility

The findings suggest that the evaluated models may have potential as pre-laboratory risk-stratification tools rather than standalone diagnostic systems. Their potential role would be to identify individuals who may warrant formal clinical and biochemical assessment, particularly in settings where laboratory testing is not immediately available or where a low-cost initial risk assessment is desirable. This interpretation is consistent with previous research exploring non-invasive ML approaches for MetS identification. However, the moderate discrimination observed in the present study does not support replacing established diagnostic criteria or biochemical assessment with the proposed models [34].

From a computational perspective, prediction latency was low once the models had been fitted, with the slowest primary-analysis model requiring less than 1 ms per participant under the tested hardware conditions. These measurements suggest that computational speed is unlikely to be the principal barrier to deployment in a comparable technical environment. Nevertheless, computational feasibility alone does not establish clinical feasibility. Data availability, calibration, interoperability, workflow integration, clinician acceptance, prospective effectiveness, and the consequences of false-positive and false-negative classifications are likely to be more important determinants of successful implementation. Current guidance similarly emphasizes that technical model performance should be distinguished from clinical usefulness and real-world validation [35].

### 4.9. Strengths and Limitations

Several methodological features strengthen the present analysis. The primary cohort retained all participants with an evaluable MetS outcome and preserved the observed outcome prevalence. Predictor eligibility was predefined to reduce outcome–predictor circularity, while preprocessing and model development were conducted within the cross-validation framework. The study also compared six classifiers under a common validation strategy and evaluated multiple complementary dimensions of model performance, including discrimination, threshold-dependent classification, calibration, decision-curve analysis, subgroup performance, multicollinearity, SHAP-based interpretability, computational runtime, and sensitivity to outcome balancing. This multidimensional evaluation provides a more comprehensive assessment than reliance on a single discrimination metric.

However, several limitations should also be considered. First, the analysis represents internal validation in a single historical dataset collected between 2012 and 2013; therefore, the reported performance may not generalize to populations with different demographic characteristics, healthcare settings, prevalence, or case mix. Cross-validation can reduce optimism within the development dataset but cannot substitute for temporal, geographical, or prospective external validation. External validation is particularly important for determining whether model discrimination and calibration are maintained when the model is applied to new populations.

Second, several behavioral variables were self-reported and may therefore be subject to recall and reporting bias. Third, BMI and body weight provide relatively simple measures of adiposity and may not capture body-fat distribution as effectively as more direct measures; waist circumference was deliberately excluded because abdominal adiposity is itself a component of the ATP III outcome definition. Fourth, the cross-sectional nature of the dataset means that the models classify prevalent MetS rather than predict future incident MetS. Fifth, the 0.50 probability threshold was selected for standardized model comparison rather than derived from a prespecified clinical decision pathway. Finally, subgroup analyses were exploratory, and the moderate overall discrimination means that the models should not be interpreted as substitutes for laboratory assessment or established diagnostic criteria.

### 4.10. Implications and Future Research

Future MetS prediction studies should define the intended prediction point before selecting predictors. A pre-laboratory screening model should be evaluated separately from a model that incorporates biochemical measurements or known cardiometabolic diagnoses. The next step for the present framework is external validation in independent populations with different prevalence and case mix, accompanied by population-specific recalibration and prespecified threshold selection.

Prospective studies should test whether model-guided referral improves identification of previously unrecognized MetS without generating an unacceptable volume of confirmatory testing. They should also evaluate whether a more parsimonious set of accessible predictors maintains performance and whether repeated measurements provide useful incremental information.

Overall, the findings indicate that accessible non-laboratory characteristics contain meaningful information for identifying individuals with prevalent MetS, but the discriminatory signal is moderate when predictors that overlap with the diagnostic definition are excluded. The results therefore support the feasibility of pre-laboratory risk stratification while also demonstrating that discrimination, threshold-dependent performance, calibration, and clinical utility provide complementary information. Further external and prospective evaluation is required before these models can be considered for routine clinical use.

## 5. Conclusions

Machine-learning models restricted to accessible non-laboratory predictors provided moderate discrimination of prevalent metabolic syndrome in this cohort. Ensemble achieved the highest mean AUC in the natural-prevalence analysis (0.751), while Random Forest and Bagging provided more balanced sensitivity and specificity at the prespecified 0.50 threshold.

Model interpretation depended materially on threshold behavior and calibration. KNN achieved high sensitivity with poor specificity, whereas AdaBoost showed very low sensitivity at 0.50 despite moderate AUC. BMI, age, and body weight contributed most strongly to the leakage-safe Random Forest predictions, with additional information from lifestyle and sociodemographic variables. These models should therefore be considered candidates for pre-laboratory risk stratification, not substitutes for established MetS diagnostic criteria. Independent external validation, recalibration, and prospective evaluation of clinically justified thresholds are required before implementation.

## Figures and Tables

**Figure 1:**
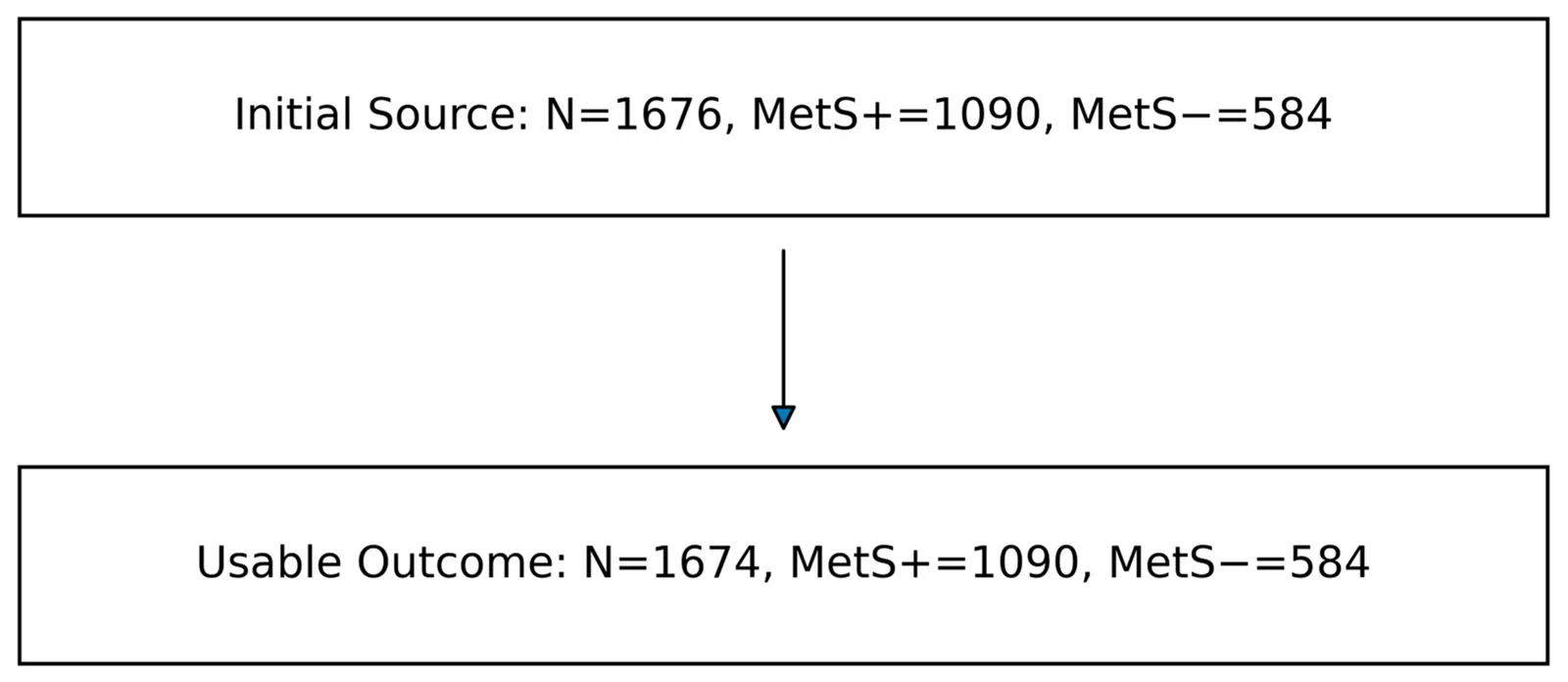
Participant Figure 1. Participant flow. Of 1676 source records, 1674 had an evaluable metabolic syndrome (MetS) outcome and were retained in the natural-prevalence primary cohort. The arrow indicates progression from the source records to the evaluable analysis cohort after exclusion of records without a usable MetS outcome.

**Figure 2:**
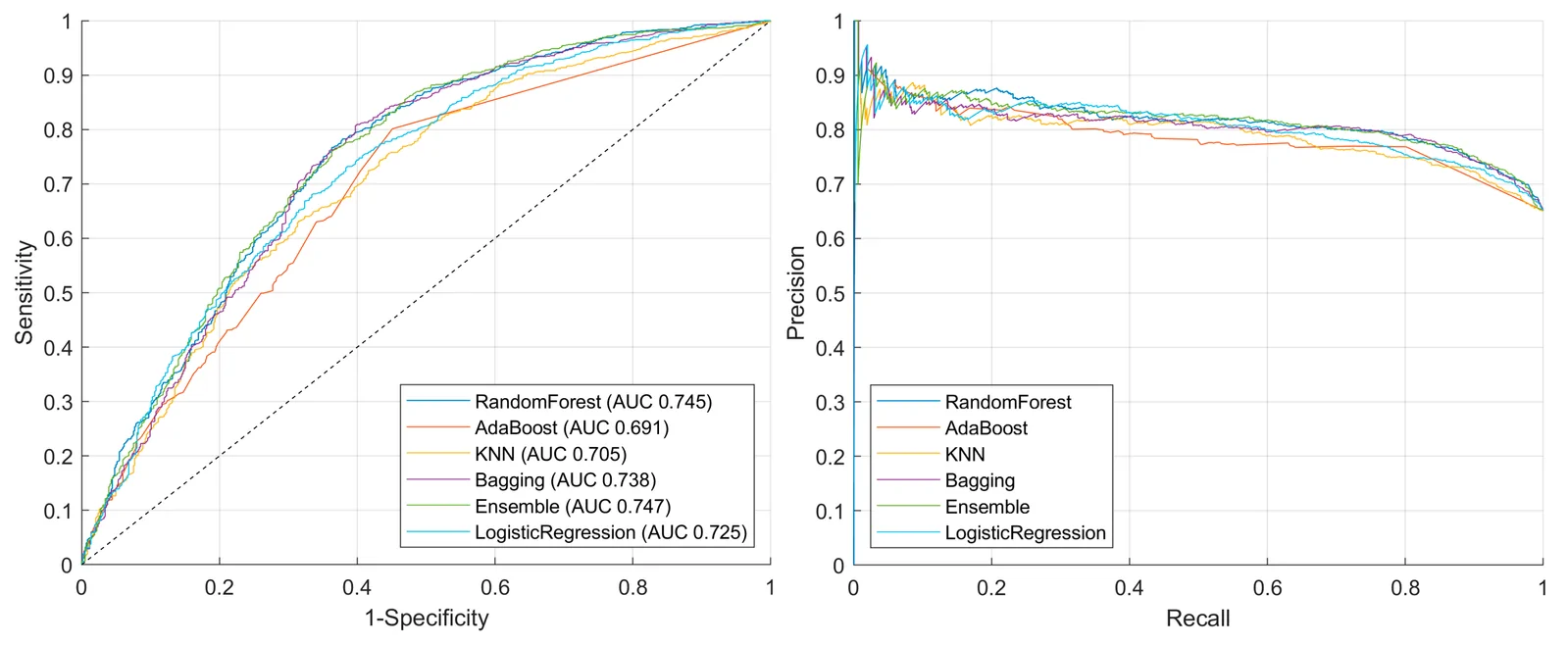
Receiver operating characteristic (ROC; **left**) and precision–recall (PR; **right**) curves generated from pooled out-of-fold predictions. AUC values displayed in the ROC panel represent pooled out-of-fold AUC estimates, whereas Table 2 reports the mean of the 10 fold-specific AUCs with 95% confidence intervals.

**Figure 3:**
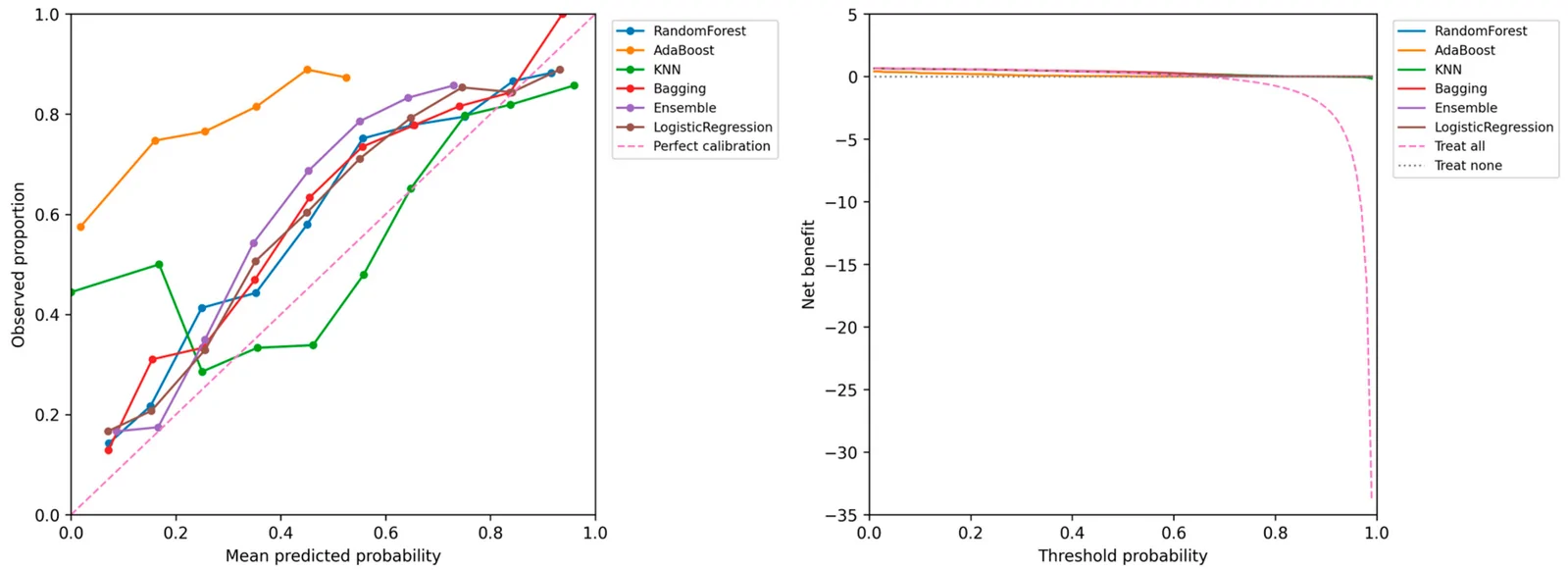
Calibration Calibration curves (**left**) and decision-curve analysis (**right**) for the primary out-of-fold predictions. In the calibration panel, the dashed diagonal line represents perfect calibration. In the decision-curve panel, model-specific net benefit is shown across threshold probabilities together with the treat-all and treat-none reference strategies.

**Figure 4:**
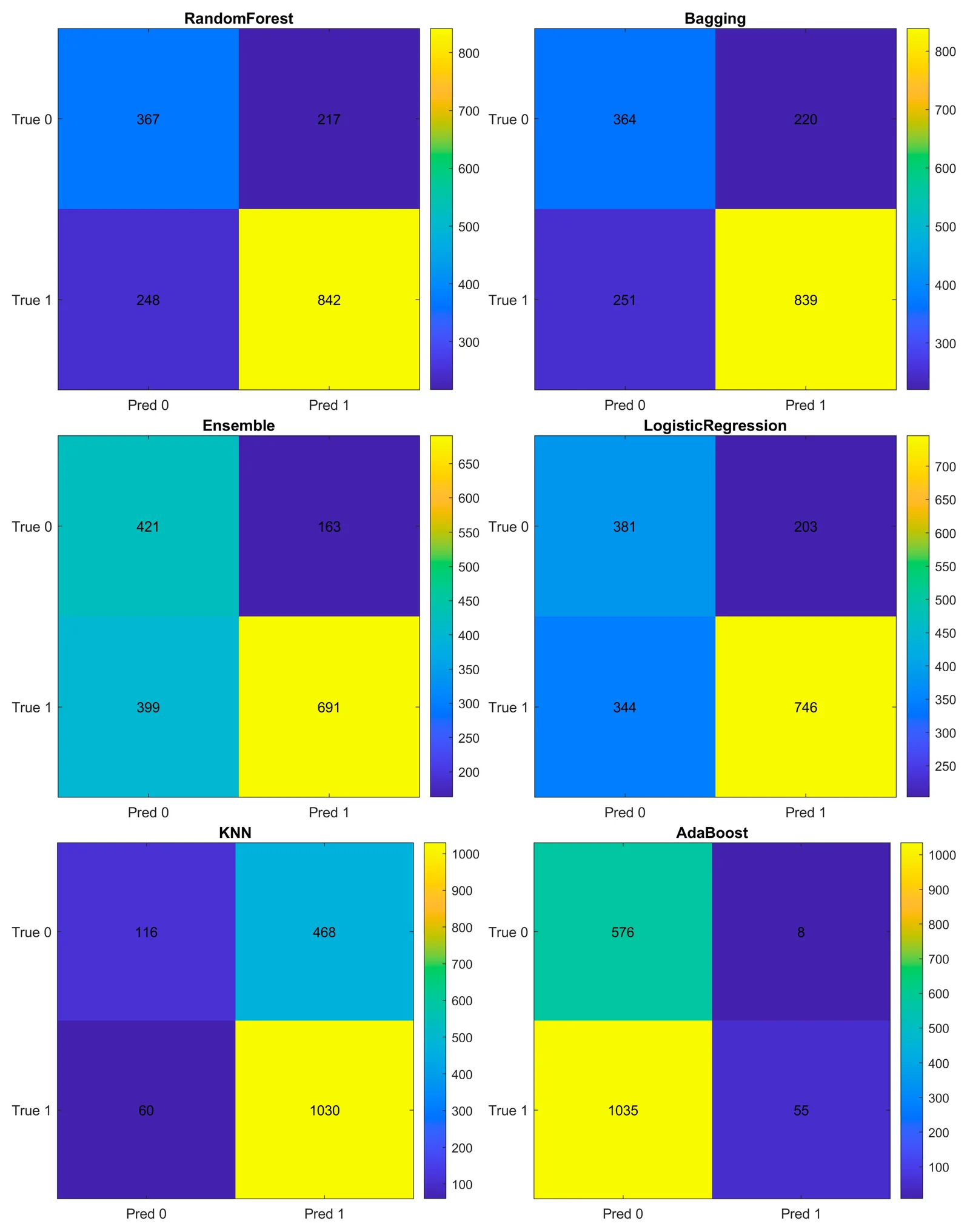
Pooled out-of-fold confusion matrices for the six classifiers in the primary analysis, using the prespecified probability threshold of 0.50.

**Figure 5:**
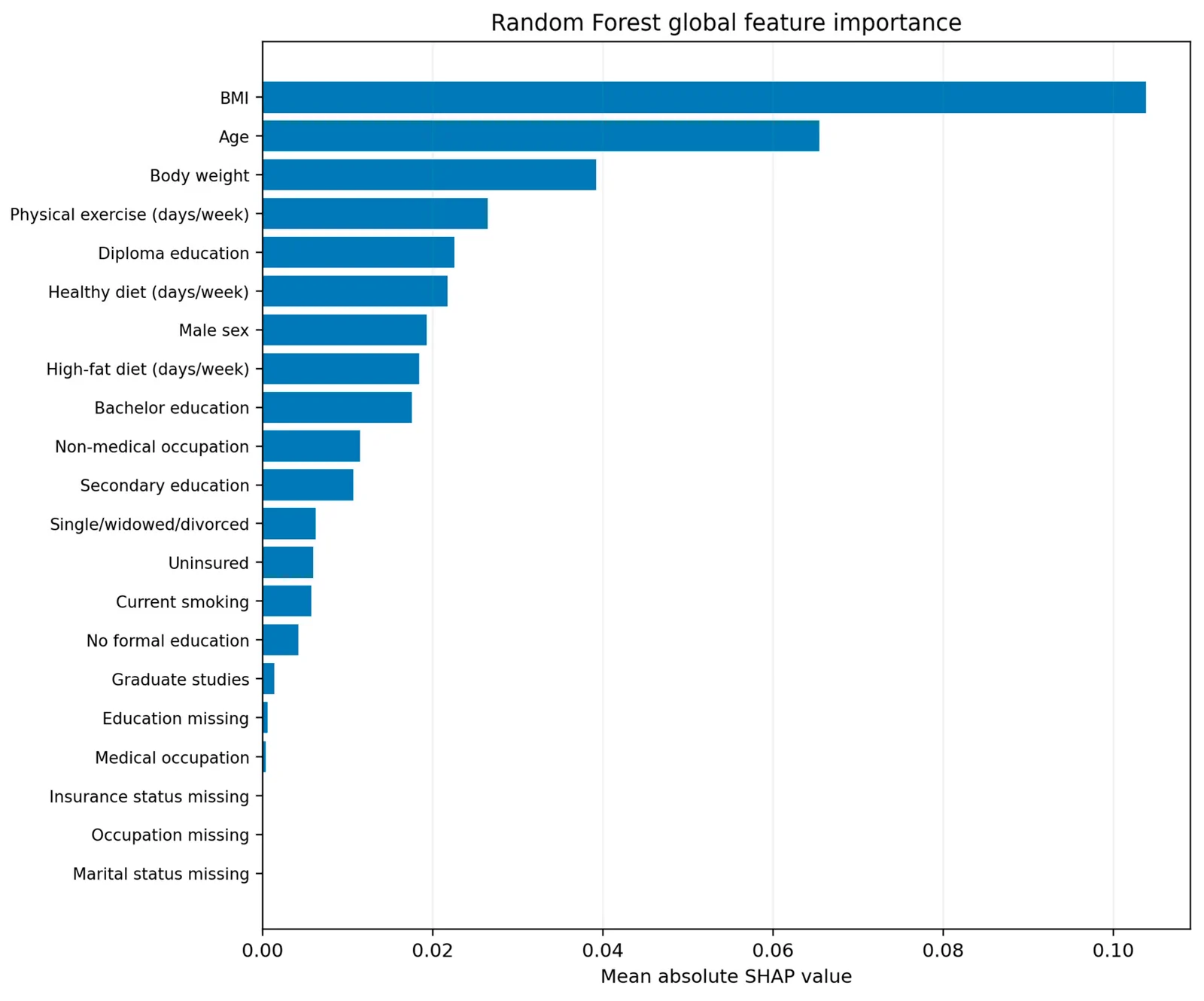
Global Random Forest feature importance expressed as mean absolute SHAP values from the final leakage-safe analysis. Labels were standardized for readability; values were calculated directly from the supplied SHAP output matrix.

**Figure 6:**
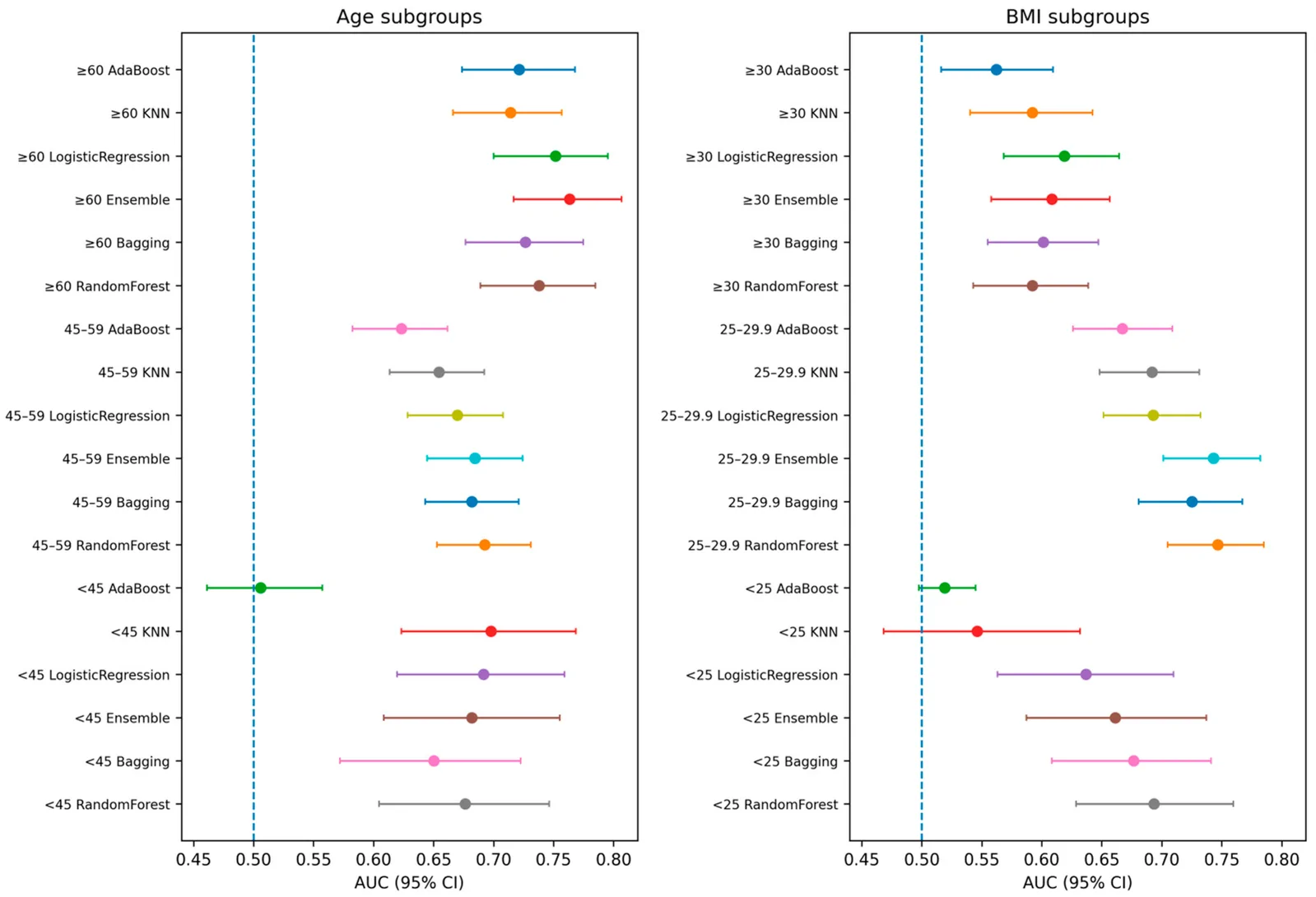
Subgroup Figure 6. Subgroup AUCs with 95% confidence intervals by age and BMI for all six classifiers in the primary analysis. Each point represents the subgroup-specific AUC for a classifier, and the corresponding horizontal line represents its 95% confidence interval. Colors distinguish classifier-specific estimates. The vertical dashed line denotes AUC = 0.50.. area under the receiver operating characteristic curve (AUC).

**Table 1:** Baseline characteristics of the natural-prevalence primary cohort.

Variable	Without MetS	MetS
Age, years	51.52 ± 10.21	56.11 ± 7.60
Body mass index, kg/m^2^	27.83 ± 4.60	30.52 ± 3.90
Body weight, kg	76.37 ± 13.48	82.88 ± 12.34
Sex: female	314 (53.8%)	678 (62.2%)
Sex: male	270 (46.2%)	412 (37.8%)
Marital status: married	509 (87.2%)	955 (87.6%)
Marital status: single/widowed/divorced	75 (12.8%)	133 (12.2%)
Education: primary	149 (25.5%)	366 (33.6%)
Education: secondary	174 (29.8%)	339 (31.1%)
Education: diploma	107 (18.3%)	125 (11.5%)
Education: bachelor	85 (14.6%)	119 (10.9%)
Education: graduate studies	14 (2.4%)	19 (1.7%)
Education: uneducated	54 (9.2%)	122 (11.2%)
Occupation: jobless	374 (64.0%)	793 (72.8%)
Occupation: non-medical job	201 (34.4%)	282 (25.9%)
Occupation: medical job	9 (1.5%)	14 (1.3%)
Health insurance: insured	554 (94.9%)	1057 (97.0%)
Health insurance: non-insured	30 (5.1%)	32 (2.9%)
Current smoker	115 (19.7%)	174 (16.0%)
Physical exercise, days/week	2.30 ± 2.97	1.91 ± 2.83
Healthy diet, days/week	2.93 ± 3.28	3.10 ± 3.25
High-fat diet, days/week	1.85 ± 2.24	2.05 ± 2.35

Data are presented as mean ± standard deviation (SD) for continuous variables and n (%) for categorical variables. Continuous variables were compared using the independent samples *t*-test, and categorical variables using the chi-square test or Fisher’s exact test, as appropriate. MetS, metabolic syndrome. AUC, area under the receiver operating characteristic curve; CI, confidence interval; SD, standard deviation.

**Table 2:** Primary cross-validated performance using the leakage-safe predictor set.

Model	Accuracy (%)	Sensitivity (%)	Specificity (%)	PPV (%)	NPV (%)	F1 (%)	AUC (95% CI)
Random Forest	72.22 ± 2.47	77.25 ± 3.45	62.86 ± 7.07	79.63 ± 2.85	59.74 ± 3.43	78.35 ± 1.98	0.745 (0.723–0.767)
Bagging	71.87 ± 3.60	76.97 ± 4.65	62.35 ± 6.53	79.29 ± 2.95	59.44 ± 5.44	78.04 ± 3.05	0.742 (0.725–0.760)
Ensemble	66.43 ± 3.97	63.39 ± 8.27	72.09 ± 6.26	81.04 ± 2.14	51.84 ± 4.40	70.83 ± 5.04	0.751 (0.731–0.771)
Logistic Regression	67.32 ± 3.72	68.44 ± 5.07	65.25 ± 5.13	78.63 ± 2.61	52.77 ± 5.05	73.10 ± 3.44	0.728 (0.705–0.751)
KNN	68.46 ± 2.30	94.50 ± 4.99	19.83 ± 10.85	68.88 ± 2.19	71.42 ± 14.62	79.57 ± 1.61	0.709 (0.685–0.733)
AdaBoost	37.69 ± 6.06	5.05 ± 11.00	98.63 ± 3.33	17.63 ± 37.20	35.93 ± 2.25	7.75 ± 16.65	0.704 (0.676–0.732)

Values except AUC are mean ± SD across the 10 outer folds. AUC values reported in Table 2 represent the mean of the 10 fold-specific AUCs, with 95% confidence intervals calculated from the corresponding fold-level AUC values. The ROC curves in Figure 2 were generated from pooled out-of-fold predictions and therefore display pooled AUC estimates. PPV, positive predictive value; NPV, negative predictive value.

**Table 3:** Calibration metrics in the primary natural-prevalence analysis.

Model	Calibration Intercept	Calibration Slope	Brier Score
Random Forest	0.532	0.915	0.195
AdaBoost	1.313	0.047	0.504
KNN	0.567	0.074	0.204
Bagging	0.581	0.917	0.198
Ensemble	0.950	1.402	0.221
Logistic Regression	0.612	1.032	0.211

**Table 4:** Performance in the 1:1 balanced sensitivity cohort.

Model	Accuracy (%)	Sensitivity (%)	Specificity (%)	PPV (%)	NPV (%)	F1 (%)	AUC (95% CI)
Random Forest	67.70 ± 5.33	72.60 ± 6.47	62.80 ± 7.00	66.21 ± 4.88	69.70 ± 6.10	69.18 ± 5.11	0.728 (0.694–0.762)
Bagging	70.30 ± 5.17	75.80 ± 5.20	64.80 ± 6.55	68.39 ± 4.98	72.81 ± 5.68	71.86 ± 4.74	0.732 (0.697–0.768)
Ensemble	64.30 ± 4.76	49.00 ± 8.01	79.60 ± 6.38	71.00 ± 7.91	61.08 ± 3.99	57.62 ± 6.78	0.742 (0.704–0.780)
Logistic Regression	66.00 ± 4.78	67.40 ± 5.58	64.60 ± 5.25	65.58 ± 4.65	66.52 ± 5.05	66.44 ± 4.86	0.711 (0.675–0.747)
KNN	63.50 ± 5.64	69.60 ± 10.78	57.40 ± 11.32	62.36 ± 5.76	66.04 ± 7.21	65.38 ± 6.15	0.700 (0.659–0.740)
AdaBoost	51.80 ± 3.05	5.00 ± 8.81	98.60 ± 2.99	25.00 ± 40.86	51.02 ± 1.75	8.04 ± 13.87	0.698 (0.660–0.736)

Values except AUC are mean ± SD across the 10 outer folds. AUC is mean with the metric-specific 95% confidence interval. AUC, area under the receiver operating characteristic curve; CI, confidence interval; SD, standard deviation; PPV, positive predictive value; NPV, negative predictive value. [Updatedmanusrcipt | Word] (https://yarmoukuniversity2014-my.sharepoint.com/personal/i_berdaweel_yu_edu_jo/_layouts/15/Doc.aspx?sourcedoc=%7BEC12D762-F1A5-45EC-AB76-7EBA455990DD%7D&file=Updatedmanusrcipt.docx&action=default&mobileredirect=true accessed on 27 July 2026).

**Table 5:** Mean computational runtime by analysis and classifier.

Analysis	Model	Training Time/Fold (s)	Prediction Time/Fold (s)	Prediction Time/Participant (ms)
Primary (N = 1674)	AdaBoost	19.28	0.0041	0.024
Primary (N = 1674)	Bagging	32.07	0.0540	0.323
Primary (N = 1674)	Ensemble	82.02	0.1502	0.897
Primary (N = 1674)	KNN	2.48	0.0094	0.056
Primary (N = 1674)	Logistic Regression	1.95	0.0008	0.005
Primary (N = 1674)	Random Forest	28.19	0.0921	0.550
Balanced sensitivity (N = 1000)	AdaBoost	20.41	0.0298	0.298
Balanced sensitivity (N = 1000)	Bagging	33.97	0.0630	0.630
Balanced sensitivity (N = 1000)	Ensemble	75.70	0.1608	1.608
Balanced sensitivity (N = 1000)	KNN	1.96	0.0068	0.068
Balanced sensitivity (N = 1000)	Logistic Regression	1.65	0.0007	0.007
Balanced sensitivity (N = 1000)	Random Forest	19.36	0.0582	0.582

Runtime measurements were generated in MATLAB R2024b on a 13th Gen Intel Core i9-13980HX computer with 32,387 MB physical memory and Microsoft Windows 11 Pro, 64-bit (build 26200). Prediction latency was small for all evaluated models in this environment; however, these measurements alone do not establish clinical real-time performance. ms, milliseconds.

## Data Availability

The datasets generated and/or analyzed during the current study are not publicly available but are available from the corresponding author upon reasonable request.

## References

[1] Gruber J., Santos B.S.d., Marção M., Durdana I., Lee B., Vumma L., Segato-Dezem F., Chasteen H., Zhang S., Lewis C. (2026). GLP-1 Targeting Agents Impair Chemoimmunotherapy Effectiveness in Triple-Negative Breast Cancer. Res. Sq..

[2] Neeland I.J., Lim S., Tchernof A., Gastaldelli A., Rangaswami J., Ndumele C.E., Powell-Wiley T.M., Després J.P. (2024). Metabolic syndrome. Nat. Rev. Dis. Prim..

[3] Saklayen M.G. (2018). The Global Epidemic of the Metabolic Syndrome. Curr. Hypertens. Rep..

[4] Noubiap J.J., Nansseu J.R., Lontchi-Yimagou E., Nkeck J.R., Nyaga U.F., Ngouo A.T., Tounouga D.N., Tianyi F.L., Foka A.J., Ndoadoumgue A.L. (2022). Geographic distribution of metabolic syndrome and its components in the general adult population: A meta-analysis of global data from 28 million individuals. Diabetes Res. Clin. Pract..

[5] Noubiap J.J., Nansseu J.R., Nyaga U.F., Ndoadoumgue A.L., Ngouo A.T., Tounouga D.N., Tianyi F.L., Foka A.J., Lontchi-Yimagou E., Nkeck J.R. (2025). Worldwide trends in metabolic syndrome from 2000 to 2023: A systematic review and modelling analysis. Nat. Commun..

[6] Ansarimoghaddam A., Adineh H.A., Zareban I., Iranpour S., HosseinZadeh A., Kh F. (2018). Prevalence of metabolic syndrome in Middle-East countries: Meta-analysis of cross-sectional studies. Diabetes Metab. Syndr. Clin. Res. Rev..

[7] Aljohani N.J. (2014). Metabolic syndrome: Risk factors among adults in Kingdom of Saudi Arabia. J. Fam. Community Med..

[8] Khader Y., Batieha A., Jaddou H., El-Khateeb M., Ajlouni K. (2010). Metabolic Syndrome and Its Individual Components among Jordanian Children and Adolescents. Int. J. Pediatr. Endocrinol..

[9] Guembe M.J., Fernandez-Lazaro C.I., Sayon-Orea C., Toledo E., Moreno-Iribas C., Cosials J.B., Reyero J.B., Martínez J.D., Diego P.G., Uche A.M.G. (2020). Risk for cardiovascular disease associated with metabolic syndrome and its components: A 13-year prospective study in the RIVANA cohort. Cardiovasc. Diabetol..

[10] Mottillo S., Filion K.B., Genest J., Joseph L., Pilote L., Poirier P., Rinfret S., Schiffrin E.L., Eisenberg M.J. (2010). The metabolic syndrome and cardiovascular risk a systematic review and meta-analysis. J. Am. Coll. Cardiol..

[11] Alberti K.G.M.M., Eckel R.H., Grundy S.M., Zimmet P.Z., Cleeman J.I., Donato K.A., Fruchart J.C., James W.P.T., Loria C.M., Smith S.C. (2009). Harmonizing the metabolic syndrome: A joint interim statement of the international diabetes federation task force on epidemiology and prevention; National heart, lung, and blood institute; American heart association; World heart federation; International. Circulation.

[12] Konuthula D., Tan M.M., Burnet D.L. (2023). Challenges and Opportunities in Diagnosis and Management of Cardiometabolic Risk in Adolescents. Curr. Diabetes Rep..

[13] Kim J.Y., Kim S.H., Cho Y.J. (2013). Socioeconomic status in association with metabolic syndrome and coronary heart disease risk. Korean J. Fam. Med..

[14] Xiaoxue W., Zijun W., Shichen C., Mukun Y., Yi C., Linqing M., Wenpei B. (2024). Risk prediction model of metabolic syndrome in perimenopausal women based on machine learning. Int. J. Med. Inform..

[15] Boisvenue J.J., Oliva C.U., Manca D.P., Johnson J.A., Yeung R.O. (2020). Feasibility of identifying and describing the burden of early-onset metabolic syndrome in primary care electronic medical record data: A cross-sectional analysis. CMAJ Open.

[16] Zhang H., Shao J., Chen D., Zou P., Cui N., Tang L., Wang D., Ye Z. (2020). Reporting and Methods in Developing Prognostic Prediction Models for Metabolic Syndrome: A Systematic Review and Critical Appraisal. Diabetes. Metab. Syndr. Obes..

[17] Esmailzadeh A., Norouzkhani N., Sezavar Dokhtfaroughi S., Rasoulian A., Mazaheri Habibi M.R. (2026). Application of Artificial Intelligence in the Diagnosis, Prediction, and Management of Metabolic Syndrome: A Systematic Review. Health Sci. Rep..

[18] Daniel Tavares L., Manoel A., Henrique Rizzi Donato T., Cesena F., André Minanni C., Miwa Kashiwagi N., Paiva da Silva L., Amaro E., Szlejf C. (2022). Prediction of metabolic syndrome: A machine learning approach to help primary prevention. Diabetes Res. Clin. Pract..

[19] Yu C.S., Lin Y.J., Lin C.H., Wang S.T., Lin S.Y., Lin S.H., Wu J.L., Chang S.S. (2020). Predicting Metabolic Syndrome With Machine Learning Models Using a Decision Tree Algorithm: Retrospective Cohort Study. JMIR Med. Inform..

[20] Li Z., Wu W., Kang H. (2024). Machine Learning-Driven Metabolic Syndrome Prediction: An International Cohort Validation Study. Healthcare.

[21] Beam A.L., Kohane I.S. (2018). Big Data and Machine Learning in Health Care. JAMA.

[22] Shin H., Shim S., Oh S. (2023). Machine learning-based predictive model for prevention of metabolic syndrome. PLoS ONE.

[23] Zhang H., Chen D., Shao J., Zou P., Cui N., Tang L., Wang X., Wang D., Wu J., Ye Z. (2021). Machine Learning-Based Prediction for 4-Year Risk of Metabolic Syndrome in Adults: A Retrospective Cohort Study. Health Policy.

[24] Trigka M., Dritsas E. (2023). Predicting the Occurrence of Metabolic Syndrome Using Machine Learning Models. Computation.

[25] Mohseni-Takalloo S., Mohseni H., Mozaffari-Khosravi H., Mirzaei M., Hosseinzadeh M. (2024). The effect of data balancing approaches on the prediction of metabolic syndrome using non-invasive parameters based on random forest. BMC Bioinform..

[26] Kakudi H.A., Loo C.K., Moy F.M., Kau L.C., Pasupa K. (2021). Diagnosis of Metabolic Syndrome Using Machine Learning, Statistical and Risk Quantification Techniques: A Systematic Literature Review. Malays. J. Comput. Sci..

[27] Barrera F.J., Brown E.D.L., Rojo A., Obeso J., Plata H., Lincango E.P., Terry N., Rodríguez-Gutiérrez R., Hall J.E., Shekhar S. (2023). Application of machine learning and artificial intelligence in the diagnosis and classification of polycystic ovarian syndrome: A systematic review. Front. Endocrinol..

[28] Park J.H., Jeong I., Ko G.J., Jeong S., Lee H. (2025). Development of a Predictive Model for Metabolic Syndrome Using Noninvasive Data and its Cardiovascular Disease Risk Assessments: Multicohort Validation Study. J. Med. Internet Res..

[29] Kim J., Mun S., Lee S., Jeong K., Baek Y. (2022). Prediction of metabolic and pre-metabolic syndromes using machine learning models with anthropometric, lifestyle, and biochemical factors from a middle-aged population in Korea. BMC Public Health.

[30] Tjoa E., Guan C. (2021). A Survey on Explainable Artificial Intelligence (XAI): Toward Medical XAI. IEEE Trans. Neural Netw. Learn. Syst..

[31] Lundberg S.M., Erion G., Chen H., DeGrave A., Prutkin J.M., Nair B., Katz R., Himmelfarb J., Bansal N., Lee S.I. (2020). From local explanations to global understanding with explainable AI for trees. Nat. Mach. Intell..

[32] Mujika A., Meier F., Steger A. (2017). Fast-Slow Recurrent Neural Networks. Proceedings of the 31st Annual Conference on Neural Information Process.

[33] Després J.P., Lemieux I. (2006). Abdominal obesity and metabolic syndrome. Nat..

[34] Xu W., Zhang Z., Hu K., Fang P., Li R., Kong D., Xuan M., Yue Y., She D., Xue Y. (2023). Identifying Metabolic Syndrome Easily and Cost Effectively Using Non-Invasive Methods with Machine Learning Models. Diabetes. Metab. Syndr. Obes..

[35] Collins G.S., Moons K.G.M., Dhiman P., Riley R.D., Beam A.L., Van Calster B., Ghassemi M., Liu X., Reitsma J.B., Van Smeden M. (2024). TRIPOD+AI statement: Updated guidance for reporting clinical prediction models that use regression or machine learning methods. BMJ.

[36] Stevens R.J., Poppe K.K. (2020). Validation of clinical prediction models: What does the “calibration slope” really measure?. J. Clin. Epidemiol..

[37] Riley R.D., Archer L., Snell K.I.E., Ensor J., Dhiman P., Martin G.P., Bonnett L.J., Collins G.S. (2024). Evaluation of clinical prediction models (part 2): How to undertake an external validation study. BMJ.

[38] Vickers A.J., Elkin E.B. (2006). Decision curve analysis: A novel method for evaluating prediction models. Med. Decis. Mak..

